# Markers of Inflammation and Fibrosis in Alcoholic Hepatitis and Viral Hepatitis C

**DOI:** 10.1155/2012/231210

**Published:** 2012-02-22

**Authors:** Manuela G. Neuman, Hemda Schmilovitz-Weiss, Nir Hilzenrat, Marc Bourliere, Patrick Marcellin, Cristhian Trepo, Tony Mazulli, George Moussa, Ankit Patel, Asad A. Baig, Lawrence Cohen

**Affiliations:** ^1^In Vitro Drug Safety and Biotechnology, MaRS Discovery Centre, 101 College Street, South Tower, Toronto, ON, Canada M5G 1L7; ^2^Department of Pharmacology and Toxicology and Institute of Drug Research, Faculty of Medicine, University of Toronto, Toronto, ON, Canada; ^3^Gastroenterology Unit, Gastroenterology Division, Rabin Medical Center, Hasharon-Golda Campus, 7 Keren Kayemet St., Petach-Tiqwa 49372, Israel; ^4^Division of Gastroenterology, Department of Medicine, Hepatitis SMBD—Jewish General Hospital Montreal, McGill University School of Medicine, 3755 Chemin de la Cote-St-Catherine, Montreal, QC, Canada, H3T 1E2; ^5^Department of Hepato-Gastroenterology, Saint-Joseph Hospital, Marseille, France; ^6^Service Hepato-Gastroenterology, Hopital Beaujon, Clichy, France; ^7^Hopital de la Croix Rousse, 103, Grande Rue de la Croix-Rousse, 69317, Lyon Cedex 04, France; ^8^Department of Microbiology, Mount Sinai Hospital and Department of Laboratory Medicine, University of Toronto, 550 University Av., Toronto, ON, Canada, M5G 1L7; ^9^Division of Gastroenterology, Sunnybrook Health Sciences Centre, Department of Medicine, University of Toronto, 2075 Bayview Ave, Toronto, ON, Canada, M4N 3M5

## Abstract

High levels of profibrinogenic cytokine transforming factor beta (TGF-**β**), metalloprotease (MMP2), and tissue inhibitor of matrix metalloprotease 1 (TIMP1) contribute to fibrogenesis in hepatitis C virus (HCV) infection and in alcohol-induced liver disease (ALD). The aim of our study was to correlate noninvasive serum markers in ALD and HCV patients with various degrees of inflammation and fibrosis in their biopsies. *Methods*. Serum cytokines levels in HCV-infected individuals in the presence or absence of ALD were measured. Student's-*t*-test with Bonferroni correction determined the significance between the groups. *Results*. Both tumor-necrosis-factor- (TNF)-**α** and TGF-**β** levels increased significantly with the severity of inflammation and fibrosis. TGF-**β** levels increased significantly in ALD patients versus the HCV patients. Proinflammatory cytokines' responses to viral and/or toxic injury differed with the severity of liver inflammation. A combination of these markers was useful in predicting and diagnosing the stages of inflammation and fibrosis in HCV and ALD. *Conclusion*. Therapeutic monitoring of TGF-**β** and metalloproteases provides important insights into fibrosis.

## 1. Introduction

Alcohol-induced liver disease (ALD) encompasses a spectrum of hepatic injury, ranging from simple steatosis to cirrhosis. Alcohol ethylic (ethanol), a hepatotoxin, produces the oldest form of liver injury known to humankind [1–4]. In addition, ethanol-inducible cytochrome P-450 (CYP2E1) increases vulnerability of the heavy drinker to commonly prescribed drugs [5–7]. Moreover, dysregulated cytokines, including TNF-*α* and downstream cytokines, play a pivotal role in the pathophysiology of ALD [8, 9]. In addition, Th1 cells produce interleukin (IL)-2, interferon (IFN)-*γ*, and TNF-*α*, that promote inflammation and cell-mediated immunity in an attempt to control infection [10–12]. Th2 cells produce IL-10 [10] and IL-4, inducing fibrogenesis [13–17]. In HCV, an impaired HCV-specific CD4^+^ T-cell response can lead to persistence of the virus characterized by inflammation [18–22]. The expression of suppressor of cytokine signaling (SOCS) proteins permits the host to better respond to therapies [23].

The initial liver histological changes are characterized by accumulation of inflammatory cells and matrix deposition around the portal vein [24–26]. In liver disease, including ALD and HCV, liver fibrosis is defined as the abnormal accumulation of extracellular matrix (ECM) [26–33]. In all cases, inflammation plays a crucial role [24, 34]. TGF-*β* mediates the effects through signal mothers against decapentaplegic (Smad) proteins [35, 36].

In response to liver injury neutrophils migrate to the site of infection through chemokines. Distinct patterns of expression of each chemokine were noted on Kupffer cells (IL-8) (CXC) [4, 36], sinusoidal endothelial cells {macrophage inflammatory protein 1 (MIP-1) (CC)} [37], hepatocytes (CXC chemokines, IL-8) [38], lymphocytes (MIP-1), (CX3C) [39], hepatic stellate cells (HSCs) {monocyte chemoattractant protein 1 (MCP-1) [40–42], regulated upon activation of normal T-cell expressed and presumably secreted (RANTES) (CC)} [42]. Other biomarkers that are attributed to HSC activity include levels of TIMPs [43, 44]. MMP-1 levels, on the other hand, significantly decreased during fibrogenesis [45]. Alcohol consumption in HCV-infected people is known to cause accelerated progression of liver fibrosis, a higher frequency of cirrhosis, and an increased incidence of HCC [46–48]. Genes associated with fibrosis/cell adhesion/ECM were not specific to alcohol and have been reported in HCV-induced liver cirrhosis [49]. 

Previously, we reported that sera levels of RANTES, TNF-*α*, IL-6, IL-8, and IL-12 as well as TGF-*β* in HCV-infected individuals are higher as compared to healthy controls [34]. A strong correlation was observed between the degree of inflammation, as shown by the histological activity index, and TNF-*α* levels, thus indicating the possibility of using TNF-*α* levels as a marker of the degree of liver inflammation. Furthermore, TGF-*β* levels were significantly higher among those with moderate and mild fibrosis (F2-F3) regardless of the inflammation, suggesting the role TGF-*β* in HCV patients takes place mostly in earlier stages of the disease before cirrhosis is well established [34].

The aim of our present study was to correlate noninvasive serum markers: cytokines, chemokines in ALD, and chronic HCV patients with various degrees of inflammation and fibrosis in their biopsies.

## 2. Methods

### 2.1. Patients

We studied 260 AH : 140 (mild histological activity index (HAI), 60, high HAI, and 60 cirrhotics; AH, comorbidity, 60 HCV (30 cirrhotics). From 1180 HCV (that declared not drinking) : 170—low fibrosis—HAI; 450—mild fibrosis—low HAI; 440—moderate fibrosis—HAI; 120—high fibrosis—high HAI. ALD was considered as resulting from long-term heavy drinking (over 80 mg/alcohol/day). The patient population was 98% Caucasian, treated in Canada, France, and Israel.

#### 2.1.1. Chemical Measurements

The laboratory services at each of the participating sites performed routine blood tests including alanine aminotransferase (ALT), aspartate amino transferase (AST), bilirubin, albumin, and platelet counts.

#### 2.1.2. Liver Biopsy

After submitting an informed consent document, all patients underwent a percutaneous liver biopsy to ascertain the diagnosis and their stage of liver injury. Biopsy specimens were fixed, paraffin-embedded, and stained with haematoxylin and eosin, Masson's trichrome, and Sirius red. All specimens were examined and graded by the pathologists of the specific medical center. A fibrosis score from 0 to 4 and an inflammatory score from 0 to 16 were adopted according to the severity and the extent of damage. Liver fibrosis was evaluated according to the METAVIR scoring system. Fibrosis (F) was staged on a scale of 0 to 4: F0, no fibrosis; F1, portal fibrosis without septa; F2, few septa; F3, numerous septa without cirrhosis; F4, cirrhosis [32].

#### 2.1.3. Inclusion/Exclusion Criteria

Each treating physician established a diagnosis based upon the following criteria: clinical presentation, a history of excessive alcohol consumption, and exclusion of other etiology, elevated liver transaminases, neutrophil counts, serum bilirubin, and impaired coagulation. In addition, diagnosis was reached using appropriate virological and histological criteria.

HCV-infected patients tested positive for antibody to HCV on third-generation enzyme-linked immunosorbent-assay (ELISA) or recombinant immunoblot assay (Abbott Laboratories, Chicago, IL, USA). After that the viral load was measured. The patients had persistently elevated serum ALT levels for more than 6 months and no evidence of infection with hepatitis B virus (absence of detectable hepatitis B surface antigen). Additionally, there was no presence of antihuman immunodeficiency virus antibodies. Also other causes of chronic liver disease (hepatotoxic drugs, autoimmune chronic hepatitis, hemochromatosis, Wilson's disease, and -1 antitrypsin deficiency) and a history of decompensated cirrhosis (ascites, bleeding esophageal varices, or hepatic encephalopathy) were excluded. Moreover, none of these patients had received immunomodulatory or antiviral therapy. Liver histology showed lesions characteristic of chronic hepatitis.

All the patients described in the study had been treated for their disease in the specific medical facility in his (her) own country. The Ethics Committees of the specific Medical Centre approved this study, which is in concordance with the ethical guidelines of the 1975 Declaration of Helsinki for research involving human participants. Informed consent was obtained from all participating patients.

#### 2.1.4. Characteristics Studied

The following characteristics were compared between the groups: sex, age, duration of HCV infection, or alcohol use (80 g or more/day for at least 1 year), liver histology, HCV genotype, and viral load. Tables 1, 2, and 3 record the characteristics of the patients studied.

#### 2.1.5. Laboratory Methods

We measured serum: IL-6, IL-8, TNF-*α*, TGF-*β*, RANTES, Fas-ligand (FAS-L), hyaluronic acid (HA), TIMP, and apoptosome (M30). We also performed the correlation between these cytokines, chemokines, and apoptosis markers with the degree of inflammation and fibrosis as shown by light microscopy (LM) as well as with other biochemical parameters such as ALT, AST, bilirubin, albumin, ferritin as well as HCV genotype, and viral load. Patient serum specimens were kept at 4°C immediately after collection, centrifuged, aliquoted for each measurement, and frozen at −80°C within 2 h of being drawn. This provides the optimal conditions for reliable results [11]. Cytoscreen Immunoassay kits (BioSource International, Camarillo, CA, USA) for human IL-6, IL-8, TNF-*α*, TGF-*β*, RANTES, TIMP quantified the cytokines as described previously [23].

### 2.2. Fas/sFasL Measurements

Cytoscreen, Immunoassay Kits, Human Fas (BioSource International, Camarillo, CA, USA), and soluble FasL (Bender MedSystems, Vienna, Austria) were used for the quantitative determination of Fas/sFasL in serum as previously described [50]. The correlation coefficient was linear (Fas *r* = 0.996; FasL *r* = 0.998) in a concentration range between 0.23 and 15 ng/mL for Fas and between 0.16 and 10 ng/mL for FasL. The samples having higher concentrations were diluted. Each specimen was analyzed in triplicate with a sensitivity and specificity of 96% and 92%, respectively. We used standards and reference reagents available from the National Institute for Biological Standards and Controls (NIBSC, Herts., UK). These methods are standardized in our laboratory according to the procedures described [11, 50, 51].

### 2.3. Apoptosis Measurements

Apoptosis was measured using the M30-Apoptosense ELISA (Bender MedSystems, Vienna, Austria) using the manufacturer instruction. It is a solid-phase, two-site immunosorbent assay. The absorbance was measured in a microplate reader at 450 nm. The correlation coefficient was linear (*r* = 0.995) in a concentration range between 50 and 1000 U/mL. The samples having higher concentrations were diluted. Each specimen was analyzed in triplicate with a sensitivity of 95% and specificity of 90%. We used standards and reference reagents available from Bender MedSystems (Vienna Austria).

### 2.4. Detection of Serum Hepatitis C Virus RNA

For quantitation of HCV RNA in human we used “AMPLICOR HCV MONITOR test (Roche Diagnostic, PQ, Canada and Neuilly, France)”. The test is specially designed for assessing viral load with the low linear sensitivity 6 × 10^2^. The procedure is based on five major steps required by the specimen preparation, reverse transcription of target RNA to generate complementary DNA (cDNA), PCR amplification of target cDNA using HCV-specific complementary primers, hybridization of the amplified DNA to oligonucleotide probes specific to the target, and detection of the probe bind amplifier. In 100 patients we have evaluated the performance of newly developed automated real-time PCR assay, the COBAS Ampliprep/COBAS TaqMan (CAP/CTM) with AMPLICOR HCV MONITOR test (Roche Diagnostic, PQ, Canada) COBAS as previously described [52]. The overall concordance for negative/positive results was 100% for HCV. All assays were equally able to quantify HCV in genotype 1. The results indicate that the real-time PCR assay covers better viral dynamic. Serum HCV RNA detection and HCV genotyping were performed for diagnostic purposes.

### 2.5. Genotyping of Hepatitis C Virus

HCV genotyping was performed at initiation of treatment in the 5′ untranslated region of the HCV genome, using reverse hybridization with the line probe assay (InGeN, Rungis, France). The HCV line-probe assay contains 15 probe lines, allowing identification of HCV types 1 to 5 as well as their subtypes a and b [23].

### 2.6. Statistical Analysis

We calculated the statistical significance of parameters by using SPSS 9.0 for windows (SPSS Inc., Chicago, Illinois, USA). Normality of the data was tested by means of Shapiro and Wilk's W-test. Most of the data was in a normal distribution. All statistical significance was assessed at the 0.05 levels. Baseline data were descriptively summarized. To test differences between groups, we compared mean and standard deviation (S.D.) of each parameter using either parametric or nonparametric tests. Differences among groups were determined by the use of confidence intervals and analysis of variance. The 2 test or Fisher's exact test was used to compare frequency data between groups. The initial histological lesions were evaluated by the non-parametric rank correlation for each parameter. Mann-Whitney and Wilcoxon rank-sum tests were used to compare values of continuous variables. Correlations between variables were analyzed calculating the Spearman rank correlation coefficient. To determine the independent prognostic value of the selected characteristics, a logistic regression model was used.

## 3. Results

### 3.1. Patient Characteristics

Baseline demographics and disease characteristics were comparable across groups (HCV. HCV-ALD, and ALD), as shown in Tables 1, 2, and 3, confirmed by the Kruskal-Wallis test. All subjects had clinically compensated but active liver disease based on evaluations of liver biopsies and ALT and AST levels. A number of laboratory abnormalities, including elevated serum aminotransferases, were seen in patients with alcoholic liver injury, in the presence or absence of HCV. In Tables 1 and 3 we reported a specific feature used to diagnose ALD patients: the AST/ALT ratio which was higher than 1.2. Ratios of AST/ALT greater than 1 are considered suggestive of alcohol-induced etiology. 

Serum AST/ALT was typically elevated 1.2–2 times in ALD individuals as well as in HCV-ALD patients. In alcoholic hepatitis, the levels of AST and ALT were 2–6 times higher than the upper limits of normal levels in healthy individuals.

Table 2 presents the characteristic analysis of HCV-patients. Consistent with previous studies and with patient populations in Europe and North America, the majority of HCV-subjects had genotype 1 and 98% of all the patients were Caucasians.

The viral load was not significantly different between the HCV (7.3 ± 6.4 × 10^6^) and ALD-HCV (6.8 ± 5.8 × 10^6^).

In patients with mild fibrosis (F3), there was a dramatic increase in the HAI score as compared to those with moderate fibrosis (F2; *P* < 0.001). Additionally, as the fibrosis progressed to high levels seen in cirrhotic patients (F4), the HAI significantly increased as compared to patients with mild fibrosis (F3; *P* < 0.05).

**Figure 1:**
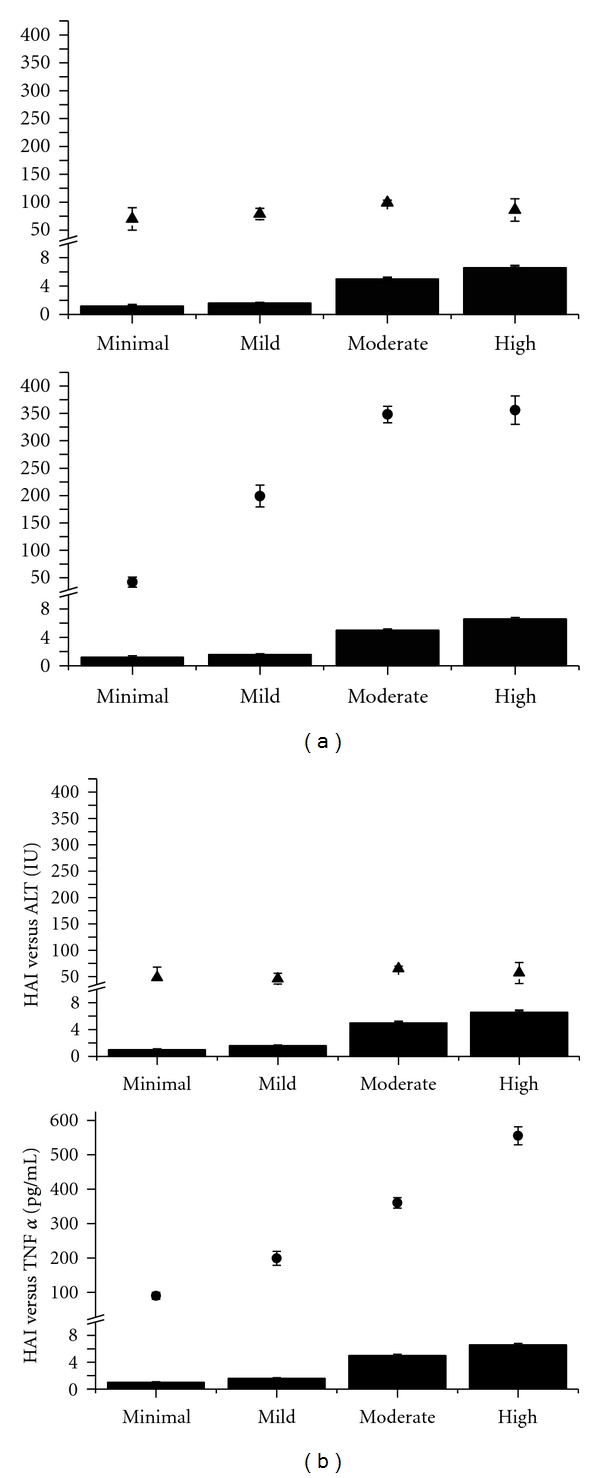
(a) Correlation between the ALT ± SD measured in U/L (upper panel) (triangles) and HAI (columns: minimal, mild, moderate, and high inflammation by histology) in HCV. The lower panel presents the correlation between TNF-*α*  ± SD ((pg/mL) rhomboid) and histological activity index (HAI-columns) in the same set of patients. (b) Correlation between the ALT (U/L ± SD (triangles)) and HAI (solid black columns) is presented in the upper panel graph. TNF-*α* ((pg/mL ± SD) rhomboid) is correlated in the lower panel with HAI (solid black columns HAI-0, mild, moderate, high) in patients with ALD.

In Figures 1(a) and 1(b), in the upper panel, the ALT values were plotted versus the HAI. As can be seen, the level of ALT was higher when compared to the normal level. However, the ALT was not significantly different in ALD patients or HCV patients in spite of the different HAI levels. Notably, there was a significant increase in TNF-*α* levels as the HAI increased (Figure 1(a) (HCV patients) and Figure 1(b) (ALD patients)). Moreover, the TNF-*α* levels in ALD patients were significantly higher (*P* < 0.05) when compared with TNF-*α* levels at the same HAI in HCV infected individuals.

Regardless the presence or absence of HCV-infection in the ALD patients, there were no significant differences between the levels of TNF-*α*. A positive correlation was found between the degree of inflammation and TNF-*α* levels (*r* = 0.90, *P* < 0.001) in all patients. Also, there was a significant increase in the levels of IL-8 and RANTES at moderate HAI as compared to patients presenting lower HAI (*r* = 0.89; *P* < 0.001), followed by a continued increase in IL-8 levels and more profoundly RANTES levels (*r* = 0.92,  *P* < 0.001). This pattern was observed in all ALD, HCV, and ALD-HCV patients. There were no significant differences between the levels of IL-8 and RANTES between the 3 types of patients. Finally, the levels of IL 6 between these individuals did not differ and were not significantly distinct when compared with the level of HAI.

As seen in the upper panel of Figure 1, ALT values do not differ with increased severity of inflammation. In contrast, as seen in the lower panel of the graph, TNF-*α* values differ significantly with the increased severity of inflammation (*P* < 0.05) up until the moderate inflammatory response. There is no significant difference in the levels of TNF -*α* between moderate and high inflammatory index. 

ALT (IU/L) values do not differ with the increased severity of inflammation neither in HCV Figure 1(a), nor in ALD individuals (Figure 1(b)). On the contrary, TNF-*α* values increase significantly with the increased severity of inflammation (*P* < 0.05) as described by a histologic inflammatory response. Both in HCV and in ALD the TNF-*α* gradually increases, paralleling the increase in HAI until the moderate HAI. There is a significant difference between the levels of TNF-*α* in high HAI versus the moderate HAI in patients with ALD (*P* < 0.05). This is an exclusive feature for the ALD individuals since in HCV-infected individuals there is a plateau of TNF-*α* (Figure 1(a)), between the patients with moderate and high HAI.

Additionally, apoptosis (M-30) increased dramatically in the moderate and high HAI. When correlating IL-8 and TNF-*α* levels with HCV genotype, no significance was observed. There was no correlation between the levels of ALT and AST with the severity of inflammation. There was a significant increase in the level of FAS-L when comparing patients with mild HAI to those with minimal HAI (*r* = 0.80, *P* < 0.05). The level of FAS-L was maintained in patients with moderate HAI, whereas in patients with high HAI a major increase was seen (*P* < 0.001). Also in ALD-HCV significant increase in FAS levels was observed in patients with moderate HAI as compared to those with minimal and mild HAI **(** *r* = 0.78, *P* < 0.05). This level was maintained in patients with high HAI. The same pattern was seen with M-30. A good correlation was observed between the apoptosome levels and TNF-*α* in patients presenting the same degree of inflammation (*r* = 0.82).

In patients with higher HAI, the levels of apoptosis and proinflammatory cytokines were significantly higher (*P* < 0.001) in patients with ALD when compared with patients with HCV.

**Figure 2:**
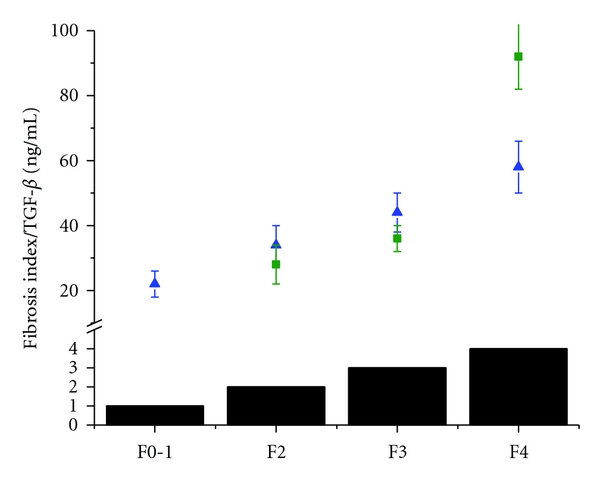
Correlation between the levels of TGF-*β* (ng/mL) in patients with HCV (triangle) and ALD (circle) and fibrosis score (solid black columns: fibrosis 0-1 (F0-1); fibrosis 2 (F2), fibrosis 3 (F3), cirrhosis (F4)).

The levels of TGF-*β* increased with the severity of liver fibrosis in HCV (Figure 2). The patients with ALD and HCV had comparable levels of TGF-*β* in the stages of fibrosis F2-F3. The ALD patients with F4 had levels of TGF-*β* two times as high as the patients with HCV. There is no statistical difference between the TGF-*β* levels of in HCV and ALD patients at F0–F3. At the highest fibrosis level (F4) there is a statistical difference (*P* < 0.05) in TGF*β* between HCV and ALD patients.

Hyaluronic acid is a glycosaminoglycan synthesized by HSCs. HA is eliminated from the circulation by sinusoidal endothelial cells. In our patients HA levels followed the same pattern as TGF-*β* in HCV, increasing significantly from  54 ± 16  (ng/mL) (F0-1) to  122 ± 32  (F2) (*P* < 0.05),  207 ± 45  (F3) (*P* < 0.05);  567 ± 85  (F4) (*P* < 0.05). In ALD patients, the levels of HA (ng/mL) also increased significantly versus the previous fibrosis stage  82 ± 16  (F2),  128 ± 25  (F3) (*P* < 0.05);  497 ± 53  (F4) (*P* < 0.001). No statistically significant difference in HA was observed between the HCV-ALD patients and the patients with non-HCV-infected ALD. Their values were  65 ± 35  (F2);  120 ± 44  (F3);  360 ± 125  (F4).

In HCV-infected individuals, it is important to observe the increase of TIMP1 values (ng/mL) (F0-1)  65 ± 12  to  126 ± 12  (F2);  147 ± 45  (F3);  672 ± 58  (F4) (*P* < 0.001 versus the previous fibrosis stages). In ALD subjects, the TIMP1 values increased from  105 ± 65  (F2),  205 ± 45  (F3),  872 ± 83  (F4) (*P* < 0.001 versus the previous fibrosis stages). The levels of TIMP1 (ng/mL) in ALD-HCV individuals were significantly different when compared with HCV individuals at the same HAI (*P* < 0.001) but not significantly different from the ALD patients.

Patients with moderate fibrosis presented a dramatic increase in the HAI as compared to those with mild fibrosis (*P* < 0.001). In addition, as the fibrosis progressed to higher levels in the cirrhotic patients, the HAI significantly increased as compared to patients with moderate fibrosis (*P* < 0.05). The levels of apoptosome (M30) were within the normal ranges in all patients that presented minimal inflammation. This level was maintained in patients with moderate HAI. There is a sequential increase in apoptosome in ALD patients. However, major increase was seen in ALD patients (*P* < 0.001) versus the moderate HAI. Additionally, in HCV individuals, a significant increase of M30 was observed (*P* < 0.05). The same pattern was seen in the levels of M30 in ALD-HCV (*P* < 0.05) increased versus the moderate HAI.

## 4. Discussion

The pattern of alcohol consumption is expressed and regulated differently in diverse geographical regions. Considering the fact that our population was recruited from Europe and North America we defined high risk drinking on the average 80 g alcohol intake/day.

Over the past fifteen years our laboratory has been focusing on the alterations of the cytokine network in patients with HCV. We have reported a correlation between TNF*α*- as a mediator of inflammation and the degree of HAI. In addition, in our previous studies with noncirrhotics, we have established that TGF*β*- levels reflect the histology fibrosis score [9]. Thus, measuring TNF*α*-and TGF*β*-levels and correlating them with HAI and fibrosis scores should bring vital information in assessing and monitoring the natural history of the disease in patients with HCV. In the present study we aimed to evaluate the cytokine-chemokine network in ALD in the presence or absence of HCV infection. Moreover, we aimed to correlate serum cytokine and chemokine levels, with the severity of the disease and to compare the same parameters in ALD patients in the presence of absence of HCV infection.

Liver fibrosis, regardless of its cause, is characterized by excess deposition of collagens and ECM proteins in the parenchyma [26]. The accumulation of interstitial collagens and ECM proteins results from an imbalance between their production and degradation by the MMPs. Fibrosis is now increasingly used as an end point for clinical trials in liver disease, as stated by the international fibrosis group [53, 54].

The tissue inhibitors of metalloproteinases designated TIMP-1, TIMP-2, and TIMP-3 regulate the extracellular activity of their MMPs, which become activated when released from the cells. TIMPs overexpression correlates with decreased activity of ECM removing MMPs. Current evidence indicates that TIMP-1 is the most important endogenous inhibitor of interstitial collagenase, whereas TIMP-2 is particularly important in the inhibition of MMP-2 [55, 56].

TIMP has also emerged as a molecule with dual action. It inhibits the activity of MMPs and prevents stellate cells apoptosis [57].

Another component of the ECM is HA, synthesized by HSCs and eliminated from the circulation by sinusoidal endothelial cells equipped with a specific receptor [58–60]. Levels of this ECM protein and other parameters of liver damage have been proposed to assess the progression of fibrosis in ALD [61, 62], as well as in patients with HCV [63]. Therefore the importance of the present work lies in defining these biomarkers and their role in ALD.

An additional aim of the present study was to evaluate several markers of fibrosis in a cohort of subjects with a primary etiology HCV and ALD in the presence or absence of HCV infection. The overall goal of this paper was to analyze in HCV and ALD possible markers of inflammation and fibrosis with focus on the ones that correlate best with the morphologic grade of inflammation and fibrosis. Moreover, special focus has been placed on three markers (TGF-*β*, TIMP1, and HA). Other scientists [64–66] also aimed at establishing tools needed to facilitate diagnosis and appropriate treatment by using circulating markers to replace some of the repeated liver biopsies currently required in the management of many patients with liver disease.

We report here for the first time detailed analyses of markers of liver inflammation and fibrosis in a subgroup of HCV and alcoholic patients whose liver biopsy was available. Although the liver biopsy has limitations, the gold standard of determining and scoring fibrosis is its histological assessment. [67]. Accordingly, a number of circulating markers of hepatic inflammation and fibrogenesis have been proposed as an alternative to this procedure. Therefore, in addition to morphologic stages of fibrosis, serum markers of fibrosis have also been considered to predict the vulnerability for the future development and progression of the fibrosis. In chronic HCV patients the combination of several tests in addition to a liver biopsy improved the diagnostic scores [68]. In addition, the concept of “rate of fibrosis progression” has been the search of noninvasive surrogate measures of liver fibrosis [69]. In ALD, Lieber's team has shown that chronic alcohol consumption may affect the levels of some of these fibrosis markers [70–72]. In addition, the role of nutrition in ALD had been emphasized [73]. In view of the present work, we consider promising candidates as noninvasive fibrosis markers: TIMP1, HA, TGF-*β*. However, the greatest clinical usefulness of this test resided in its ability to exclude cirrhosis. Better predictors of inflammation and fibrosis can be obtained by combining panels of noninvasive biomarkers in which the accuracy has been analyzed by a series of algorithms [74, 75]. A combination of new specific biomarkers like the ones we described in this paper with the well-established laboratory changes such as AST/ALT ratio [76] might help in distinguishing the etiology of hepatitis. Studies demonstrated that the presence of HCV and drinking habits are cofactors of risk for alcohol-induced liver damage [77]. Moreover, practice guidelines in treating alcoholic liver disease recognized the importance of biomarkers in identifying the seriousness of the damage [78, 79]. In addition, the genetic polymorphism in ALD was evaluated [80]. In a genome-wide association study performed in 61089 individuals, there were identified loci associated with high plasma liver enzyme in individuals with liver disease. Genes of inflammation and immunity (*CD276*, *CDH6*, *GCKR*, *HNF1A*, *HPR*, *ITGA1*, *RORA,* and *STAT4*) have been shown to be expressed in liver disease patients [81].

Our study is important, therefore, as it examines the correlation between liver inflammation and serum inflammatory markers. Serum biomarkers play a substantial role in identifying and scoring the severity of liver injury induced by alcohol and its comorbidity with HCV. These findings need to be further investigated in patient samples with biopsy-based assessment of inflammation, apoptosis, and fibrosis as we evaluate in the present cohort.

Since 98% of the entire patient population, originating from Canada, France, and Israel, was Caucasian, our study lacked diversity. Though limiting upon first glance, this aspect can actually be useful in that this population might represent a more homogeneous group regarding their genetic, immune, and environmental setting. This point was clearly demonstrated also by the recent study that investigated the role of Genotype PNPLA3 rs738409(GG) which is associated with alcoholic liver cirrhosis in alcoholic Caucasians of German ancestry [82]. In addition, excellent correlation was found between TGF-*β* and procollagen IIIN-peptide (PIIINP) and peptide in patients with alcoholic liver disease [83]. New approaches using transient elastography (Fibroscan) may add additional information to evaluate the degree of fibrosis in hepatitis C and alcohol patients [84, 85].

In summary, all of the inflammatory biomarkers were elevated both in ALD and in HCV, but some of the biomarkers that were effective for HCV did not perform as well, in a subgroup with alcoholic fibrosis as primary etiology. It should be noted that these results are based on a relatively small cohort of ALD when compared with the very large cohort of HCV.

Many individual clinical and laboratory features, along with specific histological characteristics, have also been tested as measures of disease prognosis.

Cytokines play a specific role in determining the ALD and HCV progression of liver disease. There is significant imbalance in cytokine milieu that leads to the impairment of the healing process in both ALD and HCV. This paper brings evidence suggesting that dysregulated cytokines, including TNF-*α* and downstream cytokines, play a pivotal role in the pathophysiology of ALD, HCV, and their comorbidity. Environmental factors such as the continued use of heavy alcohol can accelerate the progression of ALD disease by activating HSC and enhancing the profibrinogenic cytokine expression in the liver. However, the progression of fibrosis is highly variable, with some individuals progressing rapidly over time whereas other individuals have stable liver disease.

## Figures and Tables

**Table 1:** Baseline characteristics ALD patients.

* Characteristics *	F2 (**n** = 140)	F3 (**n** = 60)	F4 (**n** = 60)
Age (years)	27 ± 8	46 ± 12	64 ± 5*
Sex (F/M)	24/116	8/52	0/60
Alcohol consumption (years)	10 ± 4	20 ± 6	28 ± 16
ALT (U/L)	46.0 ± 3.0	65.0 ± 11.0**	57.0 ± 12.0**
AST (U/L)	45.5 ± 4.5	80.5 ± 15.0**	77.0 ± 10.0**
Bilirubin (mg/dL)	1.2 ± 0.5	1.96 ± 1.20	5.80 ± 1.50

Values represent mean ± S.D. *P < 0.05 higher than F2 and F3; **P < 0.001 higher, when compared to F2.

**Table 2:** Baseline characteristics: HCV-infected individuals.

Characteristics	F0-F1 (**n** = 170)	F2 (**n** = 450)	F3 (**n** = 440)	F4 (**n** = 120)
Age (years)	39 ± 16	47 ± 6	46 ± 7	54 ± 10*
Sex (F/M)	48/122	177/273	132/308	50/70
HCV infection (years)	15 ± 6	18 ± 7	22 ± 8	27 ± 12
ALT (U/L)	70.0 ± 20.0	79.0 ± 10.0	99.0 ± 5.0**	86.0 ± 20.0
AST (U/L)	32.0 ± 4.0	42.0 ± 8.0	40.5 ± 15.0	50.8 ± 5.0
Bilirubin (mg/dL)	0.26 ± 0.5	3.6 ± 2.5	5.5 ± 3.50	8.5 ± 5.5

Values represent means ± S.D; *P < 0.05 higher when compared to F0-F1, F2, and F3; **P < 0.001 higher when compared to F0 or F2.

**Table 3:** Baseline characteristics ALD infected with HCV.

Characteristics	F2 (**n** = 10)	F3 (**n** = 40)	F4 (**n** = 30)
Age (years)	50 ± 15	66 ± 27	64 ± 32
Sex (F/M)	0/10	2/38	5/25
HCV infection (years)	22 ± 7	25 ± 15	35 ± 25
ALT (U/L)	34.5 ± 10.5	60.5 ± 14.0*	126.0 ± 42.0**
AST (U/L)	70.0 ± 25.0	154.0 ± 45.0	250.0 ± 75.0
Bilirubin (mg/dL)	0.36 ± 0.5	5.5 ± 2.50	12.80 ± 8.00

Values represent means ± S.D; *P < 0.05 higher when compared to F2; **P < 0.001 higher when compared to F2 or F3.

## Abbreviations

ALD: Alcohol liver disease
AH: Alcoholic hepatitis
CCR/CXCR–CC/CXC: Chemokine receptor
CD: Cluster of differentiation
CXC: Chemokines presenting an amino acid between the two N-terminal cysteine residues
ECM: Extracellular matrix
ELISA: Enzyme-linked immunosorbent assay
HA: Hyaluronic acid
HAI: Hepatic activity index
HCV: Hepatitis C virus
HSC: Hepatic stellate cells
IFN: Interferon
IL: Interleukin
MCP-1: Monocyte chemo-attractant protein 1
MHC: Major histocompatible complex
MIP-1: Macrophage inflammatory protein 1
MMP: Matrix metalloproteinases
RANTES: Regulated upon activation, normal T-cell expressed, and presumably secreted
SD: Standard deviation
Smad: Signal mothers against decapentaplegic
SOCS: Suppressor of cytokine signaling
STAT: Signal transducer and activator of transcription
TIMP: Tissue inhibitor of metalloproteinases
Th: T helper
Treg: T regulatory
TGF-*β*: Transforming growth factor beta
TIMP: Tissue inhibitors of metalloproteinases
TNF-*α*: Tumour necrosis factor-alpha
